# ESTIMA, a tool for EST management in a multi-project environment

**DOI:** 10.1186/1471-2105-5-176

**Published:** 2004-11-04

**Authors:** Charu G Kumar, Richard LeDuc, George Gong, Levan Roinishivili, Harris A Lewin, Lei Liu

**Affiliations:** 1 W.M. Keck Center for Functional and Comparative Genomics, University of Illinois at Urbana-Champaign, Urbana, IL 61801, USA; 2 Department of Animal Sciences, University of Illinois at Urbana-Champaign, Urbana, IL 61801, USA

## Abstract

**Background:**

Single-pass, partial sequencing of complementary DNA (cDNA) libraries generates thousands of chromatograms that are processed into high quality expressed sequence tags (ESTs), and then assembled into contigs representative of putative genes. Usually, to be of value, ESTs and contigs must be associated with meaningful annotations, and made available to end-users.

**Results:**

A web application, Expressed Sequence Tag Information Management and Annotation (ESTIMA), has been created to meet the EST annotation and data management requirements of multiple high-throughput EST sequencing projects. It is anchored on individual ESTs and organized around different properties of ESTs including chromatograms, base-calling quality scores, structure of assembled transcripts, and multiple sources of comparison to infer functional annotation, Gene Ontology associations, and cDNA library information. ESTIMA consists of a relational database schema and a set of interactive query interfaces. These are integrated with a suite of web-based tools that allow a user to query and retrieve information. Further, query results are interconnected among the various EST properties. ESTIMA has several unique features. Users may run their own EST processing pipeline, search against preferred reference genomes, and use any clustering and assembly algorithm. The ESTIMA database schema is very flexible and accepts output from any EST processing and assembly pipeline.

ESTIMA has been used for the management of EST projects of many species, including honeybee (*Apis mellifera*), cattle (*Bos taurus*), songbird (*Taeniopygia guttata*), corn rootworm (*Diabrotica vergifera*), catfish (*Ictalurus punctatus*, *Ictalurus furcatus*), and apple (*Malus x domestica*). The entire resource may be downloaded and used as is, or readily adapted to fit the unique needs of other cDNA sequencing projects.

**Conclusions:**

The scripts used to create the ESTIMA interface are freely available to academic users in an archived format from . The entity-relationship (E-R) diagrams and the programs used to generate the Oracle database tables are also available. We have also provided detailed installation instructions and a tutorial at the same website. Presently the chromatograms, EST databases and their annotations have been made available for cattle and honeybee brain EST projects. Non-academic users need to contact the W.M. Keck Center for Functional and Comparative Genomics, University of Illinois at Urbana-Champaign, Urbana, IL, for licensing information.

## Background

Expressed sequence tag (EST) collections represent partial descriptions of the transcribed portions of genomes. They are generated from single-pass cDNA library sequencing that is often carried out by small or mid-size sequencing centers and research groups. The research may be aimed at transcriptome expression analysis using microarray, RT-PCR, or hybridization techniques. An increasing number of research centers are involved in sequencing multiple cDNA libraries. This is manifest by the exponential growth in the number of ESTs deposited to dbEST at NCBI (National Center for Biotechnology Information), and as of the May 21, 2004 update, that number is at over 21.2 million ESTs from 686 species. Small sequencing centers are faced with searching for cost-effective and convenient tools for EST management and querying, visualization, public and private user access, and functional classification.

EST processing pipelines that transform raw chromatograms into high-quality filtered sequences, and software to cluster and assemble them into contigs are convenient to implement, and various tools are now available to researchers (StackPACK [1], ESTWeb [2], ESTAP [3], PipeOnline2.0 [4]). The TIGR gene indices [5], for example, are generated by performing an all-against-all pairwise similarity search of the ESTs, and then clustered via single-node transitive closure. The clusters are fed into CAP3 [6] for assembly. We have used the following assembly protocol for in-house sequencing projects such as cattle [7] and honeybee [8]. High quality sequences are pooled and run through BlastClust [9] to form clusters of similar sequences. Then the BlastClust output is run through CAP3. Commercial software (such as Paracel [10]) that is targeted for bioinformatics centers equipped with high-performance computing machines is also available.

We describe ESTIMA (EST Information Management and Annotation) software that provides a database schema for management of raw and annotated ESTs, and is coupled with a suite of custom web-based tools that facilitate searching various aspects of ESTs and contigs, visualization, pairwise searching by BLAST [9], and functional classification based on the controlled vocabulary defined by the Gene Ontology (GO) Consortium [11]. ESTIMA accepts assembled sequences from any EST processing and clustering software, and has been equipped with password protection so that project researchers can use the ESTIMA tool confidentially with academic or industry collaborators.

To form an association between a GO term and an EST, an organism that has already been annotated with the GO terms is selected. The ESTs are searched against this reference organism's annotated sequences, and the resulting alignments are used to ascribe putative function. The ESTIMA database schema provides users the flexibility to use any reference database that has GO term association, and any number of external databases, such as NCBI's non-redundant (nr) database or EMBL's Swissprot collections, to annotate ESTs and their contigs. This flexibility in the schema is critical given the increasing number of sequenced genomes, and specialized reference databases that researchers can download and integrate with existing annotations. Other database schemas that house EST project and analysis data have been reported [3,12]. But the inherent flexibility of multiple EST project management that ESTIMA affords, by allowing users to create multiple instances of the PROJECT schema within the GENOME schema (see section on databases), and allowing any number of reference genomes to be added, is unique to ESTIMA.

The inputs to ESTIMA are the chromatograms, raw and processed ESTs, clusters and assembled EST contigs from any source, reference database annotations of ESTs, and contigs. All of these get loaded into the tables generated from the ESTIMA database schema and loading scripts. The raw and processed data, with their annotations, are made available to users through the ESTIMA query interfaces, for exporting, visualization, and further research.

## Implementation

**Figure 1:**
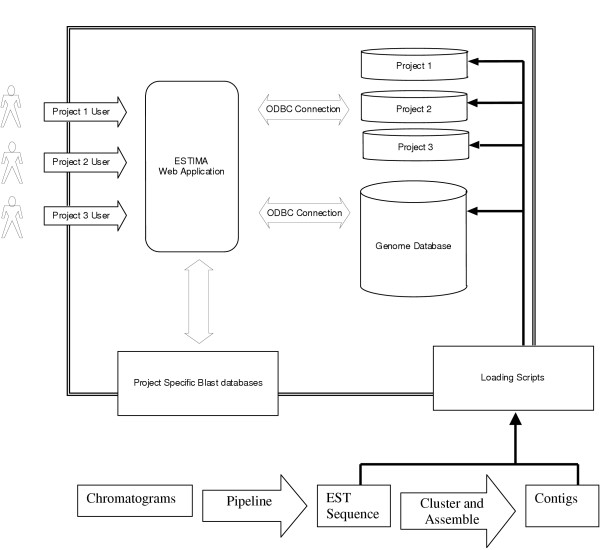
ESTIMA is organized around three major components. A single installation of the ESTIMA web application can provide a front-end for any number of projects; in this case, three different projects are shown. The web application connects to a different project database for each project. All projects share the GENOME database, and a common repository for the blastable databases, although project users can only "see" those databases associated with their project.

ESTIMA is composed of three major components: an ODBC-compliant database, loading scripts and a web application. Figure 1 shows the relationship of these components.

### Databases

**Figure 2:**
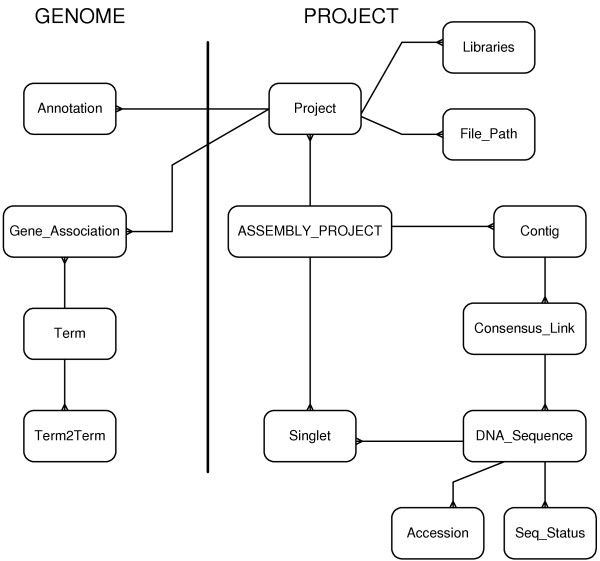
This ER diagram shows both the GENOME schema and a single PROJECT schema. In practice, each project schema is given a unique name associated with the organism under study, thus the songbird project information is stored in the "songbird" schema.

The heart of the system is a pair of database schemas. Figure 2 shows an ER diagram describing both schemas. The first schema, GENOME is common to all projects in the installation. It houses tables containing the GO structure, gene association information, annotation, and project security. GO terms are stored in two tables. Each GO term has a record in the Term table. The Term2Term table contains one record for each edge in the directional graph linking the terms. The graph can then be searched by starting with a term identifier (ID), finding all child IDs, then finding all records where each of the child terms appear as a parent. In this way, the entire graph can be searched with one call per tier below the original term.

Term_Seq_Count contains the pre-calculated counts of ESTs associated with each term, and all the terms' child terms. Pre-calculation saves time at execution, and is handled by a Perl script that is rerun each time the GO tables are updated.

The Gene_Association and Annotation table contains information that links the ESTs to the reference genomes. The Gene_Association table has one record for each reference gene that is associated with an EST or contig. The reference gene is then related to a GO term in the Term table.

GENOME also contains the Blast_All table that holds information regarding the association between the ESTs and any project-specific DNA sequences. The ESTs are searched against sequences of interest, such as NCBI's nr, and the results are parsed and loaded into the Blast_All table.

In addition to the common data, each project has its own schema that contains information such as DNA sequences, contig assemblies and paths to the chromatogram files. The PROJECT schema is centered on the Project table that contains a simple link between the other PROJECT tables and the GENOME schema. This organization has proven useful when the contents of the PROJECT schema are to be transferred directly from a sequencing facility to a Laboratory Information Management System (LIMS).

Each project may have several different contig assemblies deriving from different assembly techniques. The assemblies are all housed in the Assembly_Project, Contig, Consensus_Link and Singlet tables. Each assembly has a single ID that is used by the web application to determine which is "live". After a new assembly is completed and loaded, it can be taken live by simply changing a single number in the web applications configuration file.

Finally, the sequence data itself is stored in the DNA_Sequence table, while related GenBank accession numbers are stored in their own table. Although accession numbers are not required by the web application, if they are available, then the web application will accept them where a sequence ID would be used.

### Loading

The second major component of the ESTIMA system is a series of Perl and JAVA applications used to parse and load data into the database. Because each project will have its own needs, the loading system was not automated. Instead, several dozen separate scripts and applications have been developed to allow researchers to manipulate and analyze large data sets using standard analysis applications.

### Web application

The third component of ESTIMA is the web-application that queries and reports the EST information to the end-users.

**Figure 3:**
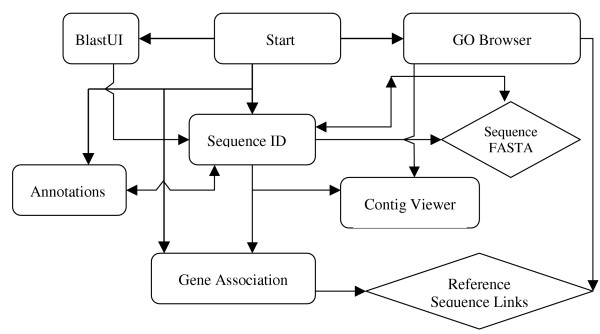
The seven elements of the web application (the start screen and six query applications shown as rectangles) interact with each other in a complex manner. A single headed arrow means that the element at the tail of the arrow creates hyperlinks in its output that automatically calls the element at the arrowhead. For example, whenever the contig viewer refers to an EST sequence, it links the ID to information about the EST from the Sequence ID element. The GO Browser and the Sequence ID elements allow users to download the appropriate FASTA files. Additionally, the GO Browser and Gene Association elements provide links to external information about reference sequences.

The ESTIMA web application is organized around seven points of entry into the system – the start screen and six query applications. Each query application interfaces with the databases, and allow users to query raw and annotated ESTs and contigs. ESTIMA supports password-protection, multiple-projects, and multiple libraries within a project. This is achieved with an XML configuration file with project-specific information that is called by the various applications when project-related information is needed. Figure 3 shows the relationship between the components of the web application. Their implementation is discussed in detail below.

#### The start screen

The start screen provides a convenient point of reference to the system. The page contains information about each library (genomic, cDNA, etc) in the project. All of the elements of the start page are contained within the configuration file.

#### GO Browser

**Figure 4:**
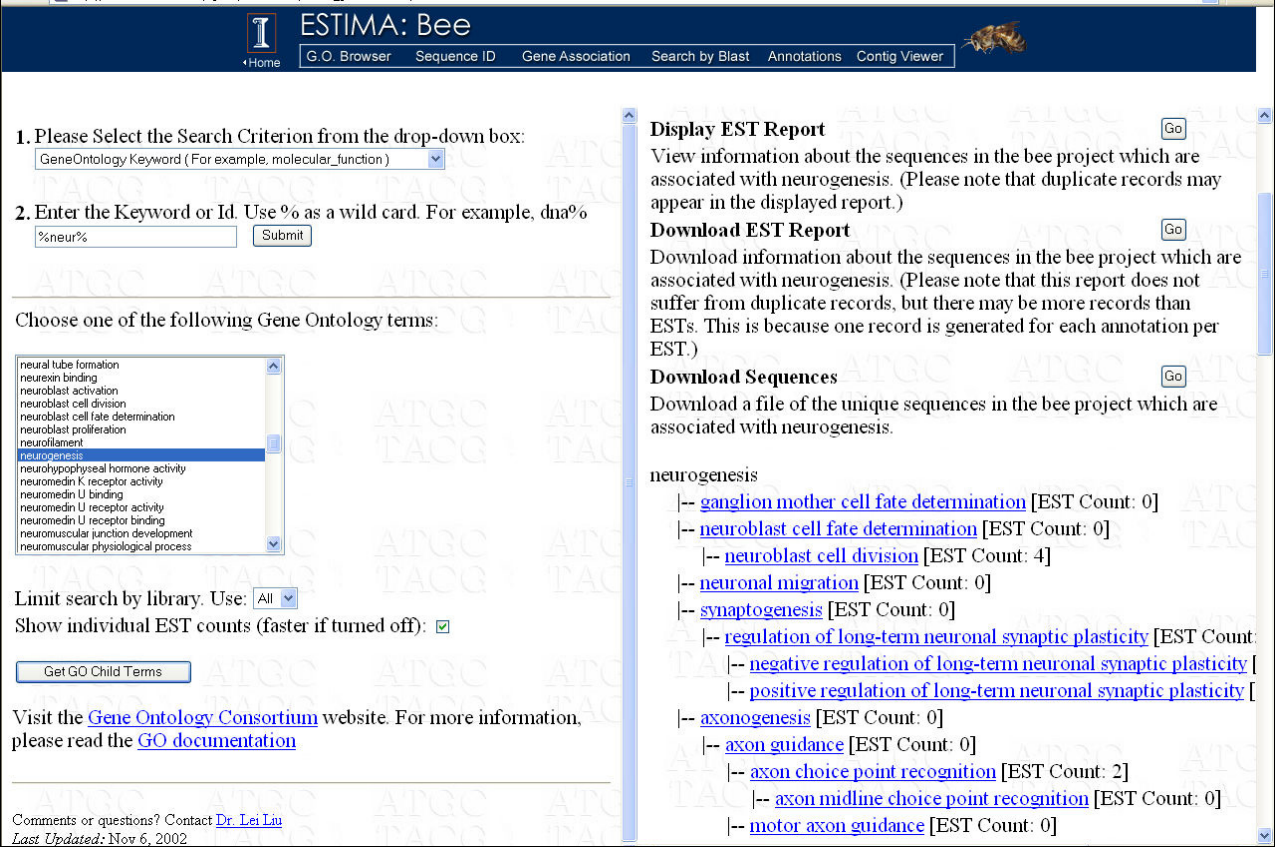
A screenshot of the custom GO Browser. The left panel is the query page, and the right panel displays the parent-term tree at the top (not visible), and a child-term tree that indicates the number of ESTs associated with each term. Detailed EST annotation reports may be displayed or downloaded, as also the sequences of these annotated ESTs.

The GO Browser (Figure 4) allows a researcher to start with a GO term, and find all ESTs associated with the term, and all of the descendent terms. The browser has a term search option that locates terms based on user-defined strings; for example, 'DNA%' will locate all GO terms starting with DNA. Once a term has been identified, the browser will provide a map of the GO tree both above and below any term, as well as a count of the ESTs associated with each of the terms displayed. For each term the browser also provides the option of either downloading the sequences of all ESTs and contigs associated with the term in a single FASTA file, or download a spreadsheet of the EST identifiers, associated GO terms and information about the linkage between the two. Alternately, the spreadsheet can be viewed as a web page. In this form the sequence identifiers will link to the appropriate ESTIMA page, ESTs to the Sequence ID page and contigs to the contig viewer. Further, the reference sequence used to form the association will link to the appropriate external web site. The GO Browser output can be filtered so that only ESTs from a single library are displayed.

#### Sequence ID

ESTs can be accessed directly from the sequence ID interface. More commonly, results from queries on annotations or contigs are dynamically linked to the EST sequence information. From these screens users can access chromatograms, and both raw and filtered sequences.

#### Gene association

Information about the association between the ESTs and the reference genome sequences can be accessed through these pages.

#### BlastUI

ESTIMA provides a Blast User Interface that allows FASTA-formatted sequences to be searched against the EST libraries. The libraries that are available for BLAST [9] can be defined for each project. We usually allow at a minimum, the raw sequences, the clean sequences, and the unique assembled sequences (contigs plus singletons). This allows researchers to rapidly identify those ESTs associated with any particular sequence of interest. The data sets available for BLAST searches can be easily extended for specific projects. For example, we make the Baylor University Honeybee Genome assemblies [13] available to the honeybee site [8].

#### Annotations

The Annotation page is an optional page, the presence or absence of which is controlled from a configuration file. This page allows additional BLAST-derived annotations, beyond GO annotations, to be displayed and queried in a number of different ways. The web application creates an interface that allows users to query the Blast_All table directly. For example, songbird ESTs [14] were searched against the nr from NCBI, Swiss-Prot, and chicken database from TIGR. A researcher can use this to find all the ESTs and contigs in the songbird collection that are associated with any term in the sequence description. The term RNA binding, for instance, returns 38 hits, of which 19 are to nr proteins, 11 to Swiss-Prot, and 8 to TIGR chicken.

#### Contig Viewer

The Contig Viewer provides an image of the assembled contigs showing the relationship of the contig with the ESTs it contains as members. It provides the contig's consensus sequence, and links to each of the member ESTs.

The web application maintains a common look and feel to all the pages within a project. This is implemented by having a single block of HTML stored within a configuration file. As each page is rendered, the same HTML is called to generate the header block of the page. Each project has its own HTML, so the look of each project's web site can be customized.

The inter-connectivity of the different ESTIMA modules allows researchers to engage in a "free form" exploration of the data. Users can query the GO Browser, for example, to find a contig associated with a term of interest, drill down to see the structure of the contig, and then, if desired, drill down to get specific information on each EST in the contig. Just as easily, the users could take a sequence of interest from their research, and similarity-search it against the assembled ESTs. Then they could drill down on an EST that aligned with their sequence, and see the additional information about the sequence, including any annotations.

### Chromatograms

ESTIMA allows users to view the actual chromatograms of the ESTs. Chromatogram files are stored in a file system visible to the web server, while file paths are stored in the database. When a user requests a chromatogram, Phred [15,16] is called to convert the chromatogram to SCF format and this is sent to Traceviewer [17] that displays the trace in the user's web browser.

## Results

### ESTIMA is independent of an EST processing pipeline

ESTIMA is unlinked from the backend EST processing pipeline, clustering, and assembly of ESTs. It serves as a stand-alone web application that allows users to store, access, research, and visualize the raw and annotated ESTs and contigs, including GO annotation. The output from a sequencing center's EST processing pipeline (base-called high-quality ESTs, assembled contigs, BLAST results against a reference genome) gets loaded into databases, and serves as input to ESTIMA. (The W.M. Keck Center will gladly share the source code to its pipeline with any interested academic institution).

Additionally, researchers may choose to use any reference genome to annotate their sequences. ESTIMA comes packaged with a flexible database schema that supports the linking of sequences to GO terms, and other user-supplied sequences. The flexibility of the ESTIMA database schema becomes more relevant with the increasing number of sequenced genomes. ESTIMA provides a flexible, password-protected, multi-project environment to researchers. It facilitates analysis of an unlimited number of ESTs and contigs linked to GO and non-GO annotations, and the download of annotated sequences related to any GO term. ESTIMA comes with an implementation of a GO browser that allows users to view the entire child term tree for any term, conveniently from a single query interface.

### There can be multiple installations of ESTIMA

ESTIMA is designed to be a stand-alone application. Each installation of the web application has all system dependent information in its own configuration files, including the information needed to connect to the databases. We maintain two instances of the interface, a development and a production copy. As new projects are introduced, they can be tested, and any interface modifications that are needed can be perfected on the development machine, concurrent with the creation of the production version of the databases. The production database can be examined using the development web application and any errors corrected before taking the system "live" to the production version.

### ESTIMA maintains multiple projects and supports multiple libraries in a project

ESTIMA is designed to accept new projects. As new EST sequencing projects are finished, they can be easily added to an existing ESTIMA installation. A new schema is created for the new project and the loading scripts are used to populate the database. All that is required to activate the web application is the addition of a block of XML code to the configuration file, and a connection string and some HTML to the system configuration file. XML tags within a configuration file control project- specific issues such as whether the data is password protected, or which BLAST databases can be accessed from the Blast User Interface.

There are three public ESTIMA projects currently administered through the W.M. Keck Center for Comparative and Functional Genomics at the University of Illinois. The bee-ESTdb [18] is a resource created from a normalized unidirectional cDNA library from which 21,408 cDNA clones were partially sequenced. These sequences were assembled into 8,966 putatively unique sequences. The contigs were then tested for similarity to sequences in the *Drosophila *genome, and based on these similarities the sequences were tentatively assigned one or more molecular functions and biological processes. Likewise, BOVEST, the cattle EST database [19], contains 17,452 cDNAs from a bovine placenta library and 6,144 from a spleen library, all of which were annotated against the human UniGene [20]. The songbird project contains 14,461 sequences from a songbird brain library [14] that were assembled into 8,526 unique sequences. ESTIMA allows multiple libraries within each project. Information about each library is stored in the configuration file, and the interface elements dynamically generate the start page and the library filters within the GO Browser.

### Practical examples that demonstrate research utility of ESTIMA

**Figure 5:**
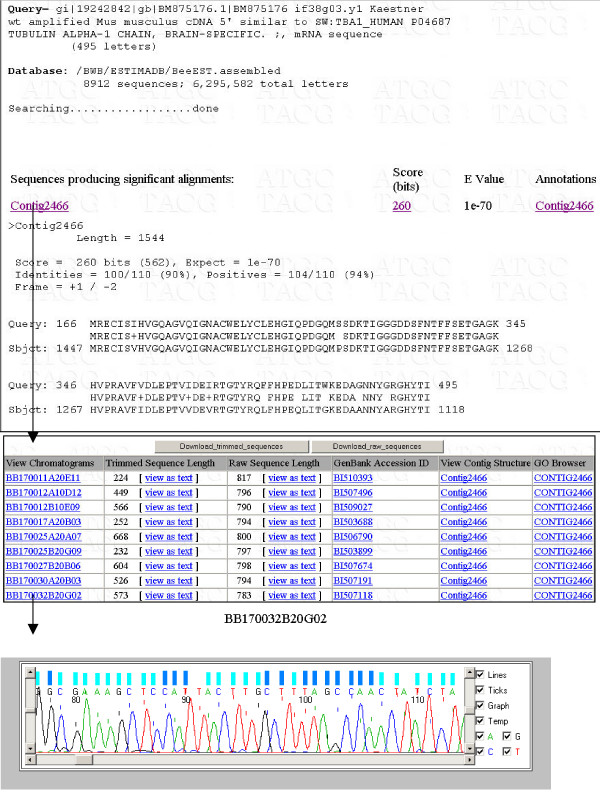
An example of the use of ESTIMA in research. The top panel shows the results of a TBLASTX of a mouse brain mRNA similar to human tubulin alpha-1 protein against honeybee brain assembled ESTs. The resulting hit Id, Contig2466, is linked to the Sequence ID interface in ESTIMA from where the consensus sequence of the honeybee contig may be retrieved. The chromatogram for a member EST in the contig is displayed.

The presence of multiple projects in ESTIMA allows for efficient cross-tissue or cross-species homology searches. For example, a mouse brain EST may be used to interrogate the honeybee brain or songbird brain library to test the hypothesis that the gene is expressed as a brain-specific transcript. Thus, a mouse brain transcript, NCBI accession number BM875176, similar to human tubulin alpha-1 chain protein may be used to do a TBLASTX against the honeybee brain assembled ESTs from the BlastUI interface in ESTIMA. The BLAST results retrieve a significant hit, Contig2466, to the honeybee brain database (Figure 5). The contig identifier is hyperlinked to the Sequence ID interface in ESTIMA from where the consensus sequence may be downloaded. The chromatograms for the ESTs that make up the contig, may also be checked for quality from the same interface in ESTIMA as shown in Figure 5. Both mouse and honeybee brain sequences, may then be used to do a deeper phylogenetic search with a BLASTX against non-redundant protein database to test the tissue-specificity hypothesis.

ESTIMA projects, as compared to other public web-applications such as TIGR gene indices [5], allow access to singlets from the EST assemblies, and chromatogram retrieval. These singlets would include rare, novel transcripts and divergent homologs that are increasingly the sole motivation for a research project. Since ESTIMA includes only high quality sequences in the databases, users may search for and download these novel transcripts, and also efficiently implement a homology search strategy using the web-application. Another strength of ESTIMA is in facilitating chromatogram and contig viewing from a common interface (Sequence ID). Any contig may be displayed and chromatograms of the member ESTs checked for errors in base-calling that may result in a premature stop-codon, or frameshift indels. Thus, ESTIMA is a valuable genome research tool.

## Conclusions

ESTIMA is a full-featured web-application and database, designed to simplify exploring and sharing EST libraries and databases. It can be easily adapted to a wide variety of system configurations, and back-end database engines. Our installation of ESTIMA easily supports three public projects, with five different EST libraries, and additionally a growing number of private projects.

## Availability and requirements

ESTIMA is available free to academic users at . Under 'Downloadable Software' section of the web page, detailed installation instructions and a user manual have been included as well. ESTIMA is still in active development. New features are constantly being developed to meet the changing needs of the research projects that use it. Further, new projects are being added to our ESTIMA installation. The system has been written to facilitate its own change, and as such, researchers should find it approachable with a good working knowledge of Perl, SQL, and HTML.

### System requirements

ESTIMA requires Perl and a database. All communication with the database is handled through Perl DBI, which is extensible to any ODBC compliant database. The use of Perl DBI and ODBC allow the databases to reside on separate servers from the web interface. Although many databases may be used, in practice, there are several complex joins in the code that could result in slow performance on large EST sets unless a well-optimized database was selected.

ESTIMA requires certain additional Perl modules, specifically BIO, CGI, DBI, and GD. Bioperl, the BIO module [21] is used extensively. All BLAST and FASTA file parsing, as well as all references to sequence objects in the user interface are handled with BIO methods. GD [22] is used to generate the Portable Network Graphics (PNG) files displayed in the contig viewer.

We have been using ESTIMA throughout its development. Our schemas are housed in an Oracle 8I database on a Silicon Graphics Origin 2000 16 × 250 MHz machine running IRIX 6.5.20. The web application, including the user requested BLAST jobs are run on a Sun Microsystems SunFire 280 R with dual 700 SPARK V9 CPUs.

## Authors' contributions

GG and LL developed the database schemas. CGK, LL and LR developed the initial web application prototype. HAL managed project development and contributed to design concepts. RL modified the web application and optimized the code. All authors read and approved the manuscript.
